# Equation Built by Multiple Adaptive Regression Spline to Estimate Biological Age in Healthy Postmenopausal Women in Taiwan

**DOI:** 10.3390/diagnostics15091147

**Published:** 2025-04-30

**Authors:** Chun-Feng Chang, Ta-Wei Chu, Chi-Hao Liu, Sheng-Tang Wu, Chung-Chi Yang

**Affiliations:** 1 Division of Urology, Department of Surgery, Tri-Service General Hospital, National Defense Medical Center, Taipei 114202, Taiwan; ccf701221@gmail.com (C.-F.C.); doc20283@gmail.com (S.-T.W.); 2 Division of Urology, Department of Surgery, Kaohsiung Armed Forces General Hospital, Kaohsiung 802301, Taiwan; 3 Department of Obstetrics and Gynecology, Tri-Service General Hospital, National Defense Medical Center, Taipei 114202, Taiwan; taweichu@gmail.com; 4 MJ Health Research Foundation, Taipei 114066, Taiwan; 5 Division of Nephrology, Department of Internal Medicine, Kaohsiung Armed Forces General Hospital, Kaohsiung 802301, Taiwan; colinliu1201@gmail.com; 6 School of Medicine, National Defense Medical Center, Taipei 114201, Taiwan; 7 Division of Cardiology, Department of Medicine, Taoyuan Armed Forces General Hospital, Taoyuan 325208, Taiwan; 8 Cardiovascular Division, Tri-Service General Hospital, National Defense Medical Center, Taipei 114202, Taiwan; 9 School of Medicine, National Tsing Hua University, Hsinchu 300044, Taiwan; 10 Institute of Bioinformatics and Structural Biology, National Tsing Hua University, Hsinchu 300044, Taiwan

**Keywords:** biological age, machine learning, postmenopausal, aging

## Abstract

**Background:** Biological age (BA) is a better representative of health status than chronological age (CA), as it uses different biological markers to quantify cellular and systemic change status. However, BA can be difficult to accurately estimate using current methods. This study uses multiple adaptive regression spline (MARS) to build an equation to estimate BA among healthy postmenopausal women, thereby potentially improving the efficiency and accuracy of BA assessment. **Methods:** A total of 11,837 healthy women were enrolled (≥51 years old), excluding participants with metabolic syndrome variable values outside two standard deviations. MARS was applied, with the results compared to traditional multiple linear regression (MLR). The method with the smaller degree of estimation error was considered to be more accurate. The lower prediction errors yielded by MARS compared to the MLR method suggest that MARS performs better than MLR. **Results:** The equation derived from MARS is depicted. It could be noted that BA could be determined by marriage, systolic blood pressure (SBP), diastolic blood pressure (DBP), waist–hip ratio (WHR), alkaline phosphatase (ALP), lactate dehydrogenase (LDH), creatinine (Cr), carcinoembryonic antigen (CEA), bone mineral density (BMD), education level, and income. The MARS equation is generated. **Conclusions:** Using MARS, an equation was built to estimate biological age among healthy postmenopausal women in Taiwan. This equation could be used as a reference for calculating BA in general. Our equation showed that the most important factor was BMD, followed by WHR, Cr, marital status, education level, income, CEA, blood pressure, ALP, and LDH.

## 1. Introduction

Aging can be defined as the gradual functional and structural decline of the human body, leading to increased vulnerability to various diseases and, eventually, death [1]. Chronological age (CA) is the duration between one’s birth to a specific later date. It is measured by days, months, and years, and it is ordinarily used to determine one’s age [2]. However, in addition to CA, an individual’s health status is subject to further lifestyle, nutritional, education, and environmental factors. Thus, CA is not a precise measurement of an individual’s physiological deterioration. Comfort was the first to propose the concept of biological age (BA), using different biological markers to quantify cellular and systemic changes during the aging process [3]. He suggested that this a better method to determine one’s health status than the CA. There have been many studies focused on this area. A vast number of publications are noted [4,5,6]. Among these studies, the most used methods were multiple logistic regression and principal component analysis [5,7,8,9].

With recent advances in artificial learning methods, machine learning (Mach-L) techniques have been widely applied in medical research [10].

Since Mach-L has two characteristics, it does not need a hypothesis as traditional statistic methods do, and it can capture non-linear relationships in the data. Mach-L has been used in many areas of medical fields. For example, artificial intelligence is applied to analyze X-rays and computer tomography scans [11,12]. At the same time, it is also used in cancer diagnosis [13], staging, and diagnosis of Parkinson’s disease [14]. It should be noted that the aforementioned studies are only the tips of the iceberg, and it is predicted that more and more applications of Mach-L will emerge in the medical fields in the future.

Unlike traditional statistical analysis methods, Mach-L does not need a hypothesis, and it is able to capture non-linear relationships within a dataset. In 1991, Freidman introduced the multiple adaptive regression spline (MARS) method, a multivariate non-parametric regression technique that can be used to build predictive equations [15]. In the past, MARS has been used to measure age-related studies, such as subadult age estimation via skeletal growth [16,17] and bone mineral density [18]. However, to our knowledge, there has been no study using MARS to estimate BA.

Since MARS can include both continuous and nominal variables and can clearly present interactions between the variables, in terms of interpretability, it provides an important advantage over other Mach-L methods, which are largely ‘black-boxes’. The present study only enrolled healthy postmenopausal women in Taiwan and applied MARS to build an equation for calculating BA for comparison with CA. Given the healthy status of the study cohort, the resulting equation could be used as a benchmark for estimating the BA of other individuals and cohorts.

## 2. Materials and Methods

### 2.1. Participant and Study Design

Following our previous work [19], the data for this study were sourced from the Taiwan MJ cohort, an ongoing prospective cohort of health examinations conducted by the MJ Health Screening Centers in Taiwan [20]. This organization is a privately operated group of three clinics that provide regular health examinations to their members. These examinations cover more than 100 important biological indicators, including anthropometric measurements, blood tests, imaging tests, etc. Each participant also completed a self-administered questionnaire to collect information of personal and family medical history, current health status, lifestyle, physical exercise, sleep habits, and dietary habits [21].

**Figure 1:**
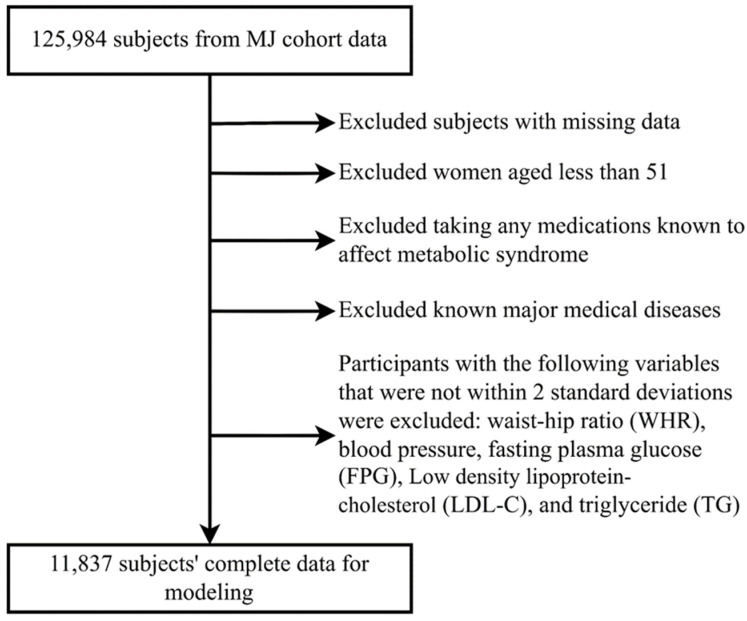
Subject identification process.

All participants signed general consent forms for future participation in anonymized studies. All participant data and physiological samples are maintained by the MJ Health Research Foundation, a non-profit affiliate of the MJ Health Screening Centers. All or part of the data used in the present study were authorized by and received from this foundation (Authorization Code: MJHRF2022009A). The study protocol was approved by the Institutional Review Board of the Kaohsiung Armed Forces General Hospital (IRB No. KAFGHIRB 111-015). Since the present study did not include any sample collection from the patients, a short review of the IRB was applied, and no additional participant consent was needed. In total, 125,984 healthy participants were enrolled. After excluding subjects for various causes and those with incomplete data, 11,837 participants remained for analysis, as shown in Figure 1.

During each health screening session, senior nursing staff recorded the subject’s medical history, including information on any current medications, and performed a physical examination. The waist circumference was measured horizontally at the level of the natural waist. The WHR was calculated based on the smallest point of the waist and the widest part of the hip. Systolic blood pressure (SBP) and diastolic blood pressure (DBP) were measured using standard mercury sphygmomanometers on the right arm of each subject while seated.

As previously published [22], the procedures for collecting demographic and biochemical data were as follows. After fasting for 10 h, blood samples were collected for biochemical analyses. Plasma was separated from the blood within 1 h of collection and stored at 30 °C until the analysis of fasting plasma glucose (FPG) and lipid profiles. FPG was measured using the glucose oxidase method (YSI 203 glucose analyzer; Yellow Springs Instruments, Yellow Springs, OH, USA). The total cholesterol and triglyceride (TG) levels were measured using the dry multilayer analytical slide method with a Fuji Dri-Chem 3000 analyzer (Fuji Photo Film, Tokyo, Japan). The serum high-density lipoprotein cholesterol (HDL-C) and low-density lipoprotein cholesterol (LDL-C) concentrations were analyzed using an enzymatic cholesterol assay, following dextran sulfate precipitation. BMD was measured by dual-energy X-ray absorptiometry (Lunar, General Electric Company, Madison, WI, USA).

Table 1 defines the 38 clinical variables (independent variables), including demographic, biochemistry, and lifestyle variables, and also presents the statistical analysis results. Drinking was defined as the multiple of total alcohol consumption duration and frequency along with alcohol content percentage. Similarly, smoking was the multiple of the duration and frequency of tobacco consumption along with the number of cigarettes consumed. Sport was the multiple of exercise duration, frequency, and type. Sleep time was an ordinal variable. Finally, age was a numerical variable, used as a dependent (target) variable.

### 2.2. Machine Learning Method

In this study, MARS was used. All methods were performed using R software version 4.0.5 [23] and RStudio version 1.1.453 [24] with the required packages installed. The implementations of MARS were the “earth” R package version 5.3.3 [25] and the “caret” R package version 6.0–94 [26]. MLR was implemented using the “stats” R package version 4.0.5, using the default settings to construct the models.

The dataset is scrutinized using MARS, a valuable approach for crafting adaptable models suited for high-dimensional data. This modeling method adopts an expansion structure reliant on product spline basis functions. Both the count of fundamental functions and the attributes connected to each, encompassing product degree and knot placements, are autonomously established through data-driven mechanisms [15]. This strategy draws inspiration from the principles of recursive partitioning, akin to methods like classification and regression trees, and mirrors its proficiency in capturing intricate higher-order interactions.

In the analysis phase, the dataset was initially partitioned into an 80% training dataset for model construction and a separate 20% testing dataset for model assessment. In the training phase, MARS uses specific hyperparameters that require tuning. To facilitate tuning, the training dataset was once more divided at random to yield a segment for model formulation using a distinct set of hyperparameters, while the other segment was used for validation purposes. A systematic exploration of all conceivable hyperparameter combinations was conducted using a comprehensive grid search approach. Subsequently, the model characterized by the lower root mean square error when applied to the validation dataset was deemed the optimal choice for each compared to MLR.

In the evaluation phase, the testing dataset was used to gauge the predictive efficacy of the MARS model. Given that the target variable in this study is a numerical parameter, the evaluation metrics chosen to compare the performance of the constructed models include relative absolute error (RAE), root relative squared error (RRSE), and root mean squared error (RMSE). The specific metric values can be found in Table 2.

## 3. Results

A total of 11,837 participants were enrolled in this study. MLR is a widely used traditional regression method so was included as a benchmark for performance comparison of the developed MARS model. The comparison results in Table 3 show that MARS yielded lower prediction errors than the MLR method.

As previously noted, MARS is particularly advantageous in this study for capturing non-linear relationships between parameters, and the hypothesis we had was that MARS is more appropriate than the traditional MLR model in estimating BA. As previously noted, MARS is particularly advantageous in this study for predicting BA. MARS is more appropriate than the traditional MLR model. In other words, MARS can provide valuable information for reference. Due to MARS’ ability to capture non-linearity in the data through the assessment of knots and the formation of basis functions, the BFs, knots, and coefficients in the MARS model are listed in Table 4. Taking BF2 and BF3, for example (both were SBP), the value of SBP was input into two equations: Max (0, 135-SBP) and Max (0, SBP-135). For each equation, the maximum value is taken into the whole equation. If the SBP is 120 mmHg, for the first equation, the result would be (0, 15), and for the second, the result would be (0, −15). According to the definition of ‘Max’, 0 would be chosen in the second equation, and −15 would be neglected.

As shown in the table, eleven key variables were selected by MARS along with the corresponding knots, for which a total of seventeen BFs with seventeen knots were acquired. Based on Table 4, the MARS equation is generated below:

$$\begin{array}{cc} BA=60.494 & -\;1.761\times Max(0,Marriage)-0.070\times Max(0,135-SBP)\\ & +0.095\times Max(0,SBP-135)-0.081\times Max(0,DBP\\ & -58)-9.012\times Max(0,0.747-WHR)\\ & +19.321\times Max(0,WHR-0.747)+0.005\times Max(0,146\\ & -ALP)-0.013\times Max(0,ALP-146)\\ & +0.008\times Max(0,LDH-274)-5.774\times Max(0,1.4\\ & -Cr)-0.693\times Max(0,Cr-1.4)-0.627\times Max(0,3.8\\ & -CEA)+39.739\times Max(0,0.647-BMD)\\ & -6.217\times Max(0,BMD-0.647)+1.473\times Max(0,2\\ & -Educationlevel)+0.629\times Max(0,2-Income)\\ & -0.157\times Max(0,Income-2) \end{array}$$

Note: SBP: systolic blood pressure; DBP: diastolic blood pressure; WHR: waist–hip ratio (%); ALP: alkaline phosphatase (U/L); LDH: lactate dehydrogenase (IU/L); Cr: creatinine (mg/dL); CEA: carcinoembryonic antigen (ng/mL); BMD: bone mass density.

In order to let the readers more easily understand and use the equation, Table 5 shows the equation in the format of an Excel file. Copy and paste this file into Excel and type all the information accordingly from A1 to A16, and the BA will be calculated in A20.

**Figure 2:**
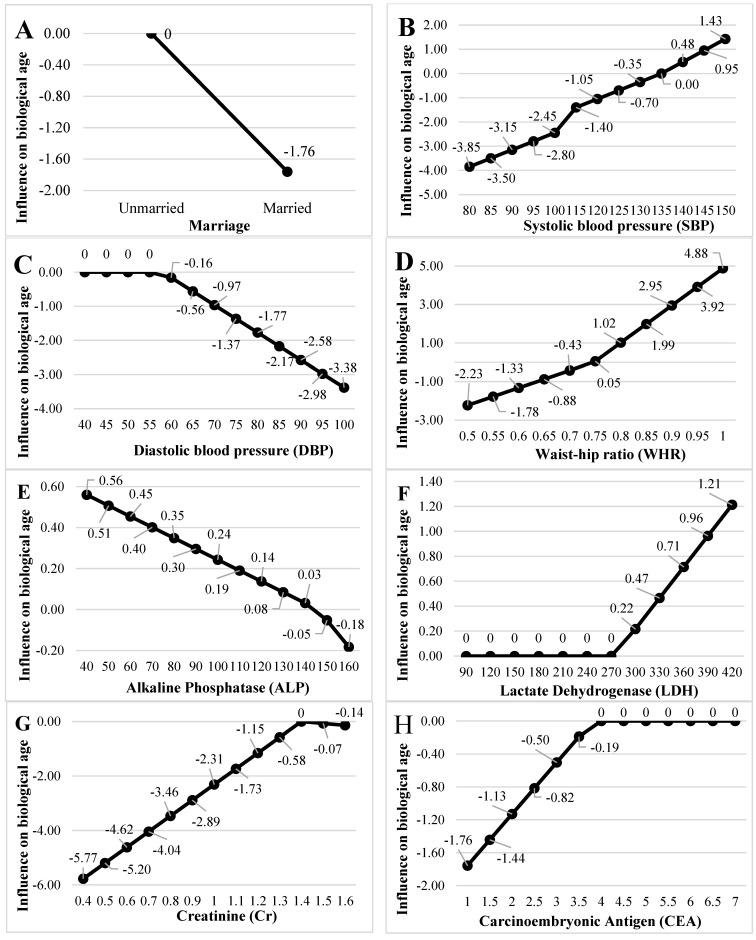

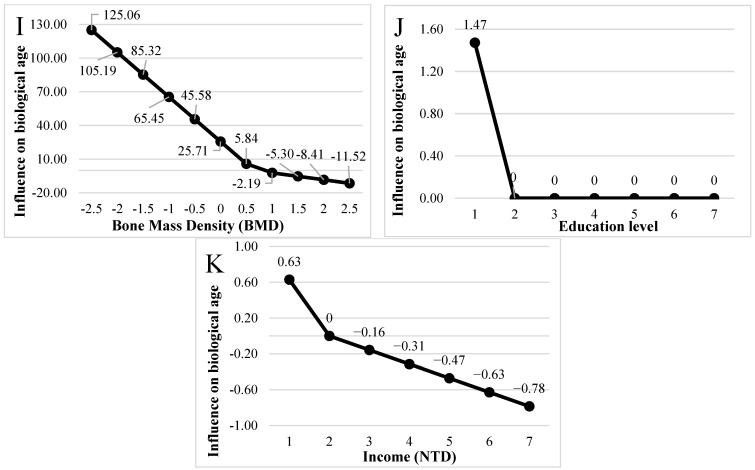
Influence of important variables on biological age. (**A**) Marriage. (**B**) Systolic blood pressure. (**C**) Diastolic blood pressure. (**D**) Waist–hip ratio. (**E**) Alkaline Phosphatase. (**F**) Lactic dehydrogenase (**G**) Creatinine. (**H**) Carcinoembryonic Antigen. (**I**) Bone Mass Density. (**J**) Education level. (**K**) Income.

To clearly understand the effect of the eleven key variables within the BF structure on BA, Figure 2 presents a visualization of the influence of the important variables on BA. Each panel in the figure features one of the important variables along with its corresponding BF. For instance, the SBP has two BFs, drawn by combining the BFs and knots of the SBP. This concept and approach were consistently applied across all panels in Figure 2, visualizing the influence of marriage, SBP, DBP, WHR, ALP, LDH, Cr, CEA, BMD, education level, and income on BA.

Due to considerations of length, we only describe Figure 2A–C. Figure 2A shows that unmarried has no impact on BA, but the BA gradually decreases after marriage. In Figure 2B, 135 mmHg is the SBP knot, and an SBP between 80 and 135 mg correlates with reduced BA, while an SBP above 135 mg correlates with increased BA. In Figure 2C, a DBP below 58 mmHg has no effect on BA, but BA decreases after the DBP exceeds 58 mmHg.

## 4. Discussion

The present study builds an equation by using MARS based on a participant group consisting of healthy postmenopausal Chinese women without the use of medication for metabolic syndrome. Participants were also excluded if they had values exceeding two standard deviations for WHR, BP, FPG, LDL-C, or TG. Thus, the resulting equation could be used widely in clinical practice to estimate biological age based on each of the included variables. For example, if a subject has higher levels for WHR, FPG, and lipids, the estimated BA should exceed the CA.

It is interesting to note that BMD had the highest coefficient among all other variables. At the same time, it is not surprising, since Xuan et al. reported that BMD is negatively correlated with age (*r* = −0.24, *p* < 0.001), aligning with accelerated biological aging in postmenopausal women [27]. Another longitudinal study of 3222 women reported a mean BMD change of −10.1% (*p* < 0.0001) [28]. The underlying pathophysiology for this relationship could be due to increased bone absorption and decreased bone formation deriving from the shift from osteoblastogenesis to mainly adipogenesis in the bone marrow. Our results are consistent with these findings, but it is surprising that BMD had the highest correlation with age.

The second important factor was WHR. In a large cohort of 40,980 postmenopausal women, Kaye et al. reported that WHR was significantly correlated with age (β = 0.003, *p* = 0.0001) after adjusting for other confounding factors [29]. Another much smaller Iranian study also found WHR to be positively related to age (r = 0.206, *p* = 0.001). WHR could be regarded as a marker for fat distribution. After menopause, there is an acceleration of fat accumulation in the waist area [30], possibly due to decreased estrogen levels, which have an effect on fat distribution [31].

Serum Cr is used as a marker for evaluating renal function. It is well known that Cr increases with increasing age. For example, Jiang et al. reported that, for women, estimated glomerular filtration rate (eGFR) declined by 1.06 mL/min/1.73 m^2^/year (95% CI: 0.99, 1.12). At the same time, for men, the decline is 0.91 mL/min/1.73 m^2^/year (95% CI: 0.86, 0.95) [32]. The equation to calculate eGFR includes age as a variable. Oo et al. modified the Gockroft–Gault equation and showed that eGFR declines approximately 1 (mL/min) per year after the age of 40 years [33]. Thus, our findings are consistent with the generally recognized relationship between age and Cr, though our study found it to be only the third most important factor.

Many previous studies have found that subjects whose reported marital status is never married, divorced, or widowed have higher mortality rates than married subjects [34,35,36]. The longevity of married persons could be explained by two reasons. First, individuals tend to select low-risk individuals as marital partners [37]. Secondly, marriage can provide protective effects for the individual [38]. It is interesting to note that one key reason for this phenomenon is health care utilization toward the end of life, since the health care expense increases continuously [39]. However, this conclusion might not be suitable to explain the findings of the present study, since the cost of health care in Taiwan is relatively low due to the country’s national healthcare program. Other more reasonable causes are likely to include ‘marriage protection’ effects, such as increased social support and income; reduced risky behavior; and reduced stress.

All other factors in our equation have been reported to be related to age, including education level [9], income [40], CEA [41,42], blood pressure [43], ALP [44], and LDH [45]. Since all of these factors had coefficients less than 1, they are less important in this equation and, thus, are not discussed in detail. The present study is subject to certain limitations. First, the proposed equation includes many complicated methods and uncommon laboratory data, such as BMD or CEA, and thus, application is limited to subjects who are able to provide such data. Secondly, the present study was only performed on ethnic Chinese, and thus, extrapolation of our findings to other ethnic groups should be performed with caution.

## 5. Conclusions

MARS was used to build an equation for estimating BA in a group of postmenopausal Chinese women. To ensure the equation’s accuracy, outliers with certain factors within two standard deviations were excluded. Our equation showed that the most important factor for determining BA was BMD, followed by WHR, Cr, marriage, education level, income, CEA, blood pressure, ALP, and LDH. The proposed equation could be used to estimate BA as a comparison to CA.

## Figures and Tables

**Table 1:** Demographic data of participants.

Ordinal Variable (Unit)		N (%)	Interval Variable (Unit)	Mean ± SD
Education level	Illiterate	2663 (22.50%)	WBC: White blood cells (×10^3^/μL)	5.85 ± 1.52
	Elementary school	5118 (43.24%)	Hb: Hemoglobin (g/dL)	13.24 ± 1.07
	Secondary	1245 (10.52%)	Plt: Platelets (×10^3^/μL)	234.99 ± 57.95
	High school	1462 (12.35%)	FPG: Fasting plasma glucose (mg/dL)	97.85 ± 9.60
	College	636 (5.37%)	TB: Total bilirubin (mg/dL)	0.74 ± 0.29
	The University	600 (5.07%)	Alb: Albumin (g/dL)	4.41 ± 0.26
	Graduate School	113 (0.95%)	Glo: Globulin (g/dL)	3.18 ± 0.39
Marriage	Unmarried	2913 (24.61%)	ALP: Alkaline phosphatase (U/L)	152.47 ± 56.17
	Married	8924 (75.39%)	SGOT: Serum glutamic-oxaloacetic transaminase (IU/L)	24.66 ± 15.62
Income (NTD)	≤200,000	6501 (54.92%)	SGPT: Serum glutamic-pyruvic transaminase (IU/L)	23.92 ± 23.49
	200,001–400,000	3010 (25.43%)	r-GT: Gamma glutamyl transpeptidase (IU/L)	19.88 ± 22.86
	400,001–800,000	1492 (12.60%)	LDH: Lactate dehydrogenase (IU/L)	323.02 ± 78.09
	800,001–1,200,000	599 (5.06%)	Cr: Creatinine (mg/dL)	0.84 ± 0.28
	1,200,001–1,600,000	132 (1.12%)	UA: Uric acid (mg/dL)	5.50 ± 1.32
	1,600,001–2,000,000	47 (0.40%)	TG: Triglycerides (mg/dL)	110.58 ± 43.37
	>2,000,000	56 (0.47%)	HDL-C: High-density lipoprotein- cholesterol (mg/dL)	58.58 ± 13.73
Sleep time (hours)	<4	NA	LDL-C: Low-density lipoprotein- cholesterol (mg/dL)	128.78 ± 29.08
	4–6	335 (2.83%)	Ca: plasma calcium level (mg/dL)	9.24 ± 0.41
	6–7	3476 (29.37%)	P: plasma phosphate level (mg/dL)	3.73 ± 0.45
	7–8	7067 (59.70%)	AFP: Alpha-fetoprotein (ng/mL)	3.40 ± 10.31
	8–9	959 (8.10%)	CEA: Carcinoembryonic antigen (ng/mL)	1.76 ± 5.97
	>9	NA	TSH: Thyroid-stimulating hormone (μIU/mL)	1.82 ± 3.43
			CRP: C-reactive protein (mg/dL)	0.25 ± 0.55
Age (years)		57.96 ± 6.50	FEV1: Forced expiratory volume in one second	1.65 ± 0.41
SBP: Systolic blood pressure (mmHg)		126.37 ± 16.54	BMD: Bone mass density	0.58 ± 0.11
DBP: Diastolic blood pressure (mmHg)		73.57 ± 9.49	Drink area	0.91 ± 6.09
WHR: Waist–hip ratio (%)		0.80 ± 0.06	Smoke area	0.87 ± 6.00
PR: Pulse rate (time/min)		72.58 ± 9.86	Sport area	6.05 ± 8.05
RR: Respiratory rate (time/min)		17.54 ± 1.52		

**Table 2:** Equations for calculating performance metrics.

Metric	Description	Calculation
RAE	Relative absolute error	$RAE=\sqrt{\frac{\sum_{i=1}^{n}{\left(y_{i}-{\hat{y}}_{i}\right)}^{2}}{\sum_{i=1}^{n}{\left(y_{i}\right)}^{2}}}$
RRSE	Root relative squared error	$RRSE=\sqrt{\frac{\sum_{i=1}^{n}{\left(y_{i}-{\hat{y}}_{i}\right)}^{2}}{\sum_{i=1}^{n}{\left(y_{i}-\bar{y_{\dot{i}}}\right)}^{2}}}$
RMSE	Root mean squared error	$RMSE=\sqrt{\frac{1}{n}\sum_{i=1}^{n}{\left(y_{i}-{\hat{y}}_{i}\right)}^{2}}$

${\hat{y}}_{i}$ and $y_{i}$ represent predicted and actual values, respectively; n stands for the number of instances.

**Table 3:** The results of equations for calculating performance metrics.

Methods	RAE	RRSE	RMSE
MARS	1.234	1.263	7.879
MLR	1.253	1.411	8.805

**Table 4:** Basis functions and important variables of the MARS model.

Corresponding Equations of the Model		
	Equation	Coefficients
Intercept	-	60.494
BFs		
BF1	Max(0, Marriage)	−1.761
BF2	Max(0, 135-SBP)	−0.070
BF3	Max(0, SBP-135)	0.095
BF4	Max(0, DBP-58)	−0.081
BF5	Max(0, 0.747-WHR)	−9.012
BF6	Max(0, WHR-0.747)	19.321
BF7	Max(0, 146-ALP)	0.005
BF8	Max(0, ALP-146)	−0.013
BF9	Max(0, LDH-274)	0.008
BF10	Max(0, 1.4-Cr)	−5.774
BF11	Max(0, Cr-1.4)	−0.693
BF12	Max(0, 3.8-CEA)	−0.627
BF13	Max(0, 0.647-BMD)	39.739
BF14	Max(0, BMD-0.647)	−6.217
BF15	Max(0, 2-Education level)	1.473
BF16	Max(0, 2-Income)	0.629
BF17	Max(0, Income-2)	−0.157

Note: Education level stage—Illiterate = 1, Elementary school = 2, Secondary = 3, High school = 4, College = 5, The University = 6, Graduate School = 7. Income stage—≤ 200,000 = 1; 200,001–400,000 = 2; 400,001–800,000 = 3; 800,001–1,200,000 = 4; 1,200,001–1,600,000 = 5; 1,600,001–2,000,000 = 6; >2,000,000 = 7. The equation is the hinge function, which takes the form of max(0,variable−knot) or $\max\left(0,knot-variable\right)$. SBP: systolic blood pressure; DBP: diastolic blood pressure; WHR: waist–hip ratio (%); ALP: alkaline phosphatase (U/L); LDH: lactate dehydrogenase (IU/L); Cr: creatinine (mg/dL); CEA: carcinoembryonic antigen (ng/mL); BMD: bone mass density.

**Table 5:** The details of the equation in the Excel file.

	A	B	C
1	Type Marriage	=MAX(0, A1)	=−1.761 × B1
2	Type SBP	=MAX(0, 135-A2)	=−0.070 × B2
3		=MAX(0, A2-135)	=0.095 × B3
4	Type DBP	=MAX(0, A4-58)	=−0.081 × B4
5	Type WHR	=MAX(0, 0.747-A5)	=−9.012 × B5
6		=MAX(0, A5-0.747)	=19.321 × B6
7	Type ALP	=MAX(0, 146-A7)	=0.005 × B7
8		=MAX(0, A7-146)	=−0.013 × B8
9	Type LDH	=MAX(0, A9-274)	=0.008 × B9
10	Type Cr	=MAX(0, 1.4-A10)	=−5.774 × B10
11		=MAX(0, A10-1.4)	=−0.693 × B11
12	Type CEA	=MAX(0, 3.8-A12)	=−0.627 × B12
13	Type BMD	=MAX(0, 0.647-A13)	=39.739 × B13
14		=MAX(0, A13-0.647)	=−6.217 × B14
15	Type Education level	=MAX(0, 2-A15)	=1.473 × B15
16	Type Income level	=MAX(0, 2-A16)	=0.629 × B16
17		=MAX(0, A16-2)	=−0.157 × B17
18			
19	BA		
20	=60.494 + SUM(C1:C17)		

Note: SBP: systolic blood pressure; DBP: diastolic blood pressure; WHR: waist–hip ratio (%); ALP: alkaline phosphatase (U/L); LDH: lactate dehydrogenase (IU/L); Cr: creatinine (mg/dL); CEA: carcinoembryonic antigen (ng/mL); BMD: bone mass density.

## Data Availability

Data are available on request due to privacy/ethical restrictions.
